# Development of a machine learning model for hepatic steatosis screening using non-invasive Traditional Chinese Medicine diagnostics and clinical variables: a health checkup study with community screening potential

**DOI:** 10.3389/fmed.2025.1704441

**Published:** 2026-01-14

**Authors:** Ke Zhu, Lihua Li, Zhihui Zhao, Sheng Zheng, Bing Lin, Wenjun Tang, Weihong Li

**Affiliations:** 1 Department of Traditional Chinese Medicine Orthopedics and Traumatology, The Third Affiliated Hospital, Southern Medical University, Guangzhou, Guangdong, China; 2 School of Basic Medical Sciences, Chengdu University of Traditional Chinese Medicine, Chengdu, Sichuan, China; 3 Department of Traditional Chinese Medicine, China-Singapore Guangzhou Knowledge City Hospital, Guangzhou, Guangdong, China; 4 School of Intelligent Medicine, Chengdu University of Traditional Chinese Medicine, Chengdu, Sichuan, China; 5 School of Rehabilitation Medicine, Gannan Medical University, Ganzhou, Jiangxi, China; 6 Healthcare Management Center, Hospital of Chengdu University of Traditional Chinese Medicine, Chengdu, Sichuan, China; 7 Department of Respiration, Hospital of Chengdu University of Traditional Chinese Medicine, Chengdu, Sichuan, China; 8 School of Traditional Chinese Medicine, Sichuan College of Traditional Chinese Medicine, Mianyang, Sichuan, China

**Keywords:** hepatic steatosis, machine learning, non-invasive screening, Traditional Chinese Medicine, XGBoost

## Abstract

**Background:**

Steatotic liver disease (SLD), underpinned by hepatic steatosis, is a global health concern affecting approximately 30% of the population. Current screening methods primarily rely on laboratory tests and lack broad-spectrum applicability. This study aims to develop a predictive model by selecting from non-invasive Traditional Chinese Medicine (TCM) diagnostics, demographic, and anthropometric variables to enhance early detection of hepatic steatosis.

**Methods:**

Data from 1,703 local residents undergoing health checkup at the health management center of Affiliated Hospital of Chengdu University of Traditional Chinese Medicine between December 2018 and December 2021 were analyzed. Demographic, anthropometric, and TCM diagnostic data were collected using questionnaires and standardized instruments. Hepatic steatosis was diagnosed via ultrasonography. Predictive models were developed using three parametric and six non-parametric algorithms, evaluated through nested five-fold stratified cross-validation. Performance was evaluated in terms of discrimination, classification metrics at the optimal threshold, calibration, and clinical utility.

**Results:**

Anthropometric variables body mass index (BMI), weight, diastolic blood pressure, and TCM diagnostic indicators HSV_H of nose, T5, phlegm-dampness constitution score, RGB_R of mid tongue, Lab_A of lip, T4, H5, and Lab_A of orbit, a total of 11 variables were selected as predictors. Logistic regression (AUC 0.83, 95% CI: 0.809–0.850) and XGBoost (AUC 0.84, 95% CI: 0.818–0.859) achieved the highest AUC among parametric and non-parametric models, respectively. XGBoost showed marginally better performance than logistic regression in AUC and clinical utility. Difference of classification metrics, calibration slops, and calibration intercepts of the two models was not statistically significant. SHAP analysis identified BMI and body weight as the most influential predictors, alongside substantial contributions from TCM features (HSV_H of nose and T5).

**Conclusion:**

TCM features combined with anthropometric variables can be used to develop a non-invasive screening model for ultrasound-diagnosed hepatic steatosis. Both the XGBoost and Logistic Regression models demonstrated robust performance, though external validation is needed to confirm generalizability. This non-invasive approach offers a practical tool with potential for hepatic steatosis screening in community settings.

## Introduction

Steatotic liver disease (SLD, formerly named fatty liver disease), characterized by excessive lipid accumulation in hepatocytes, represents a growing global health burden encompassing metabolic dysfunction-associated steatotic liver disease (MASLD), metabolic dysfunction and alcohol-associated steatotic liver disease (MetALD), alcohol-associated liver disease (ALD), and other subtypes (1). Collectively, these conditions affect approximately 30% of the global population, with regional prevalence ranging from 25% in Western Europe to 44% in Latin America, driven primarily by obesity, type 2 diabetes, and alcohol consumption (2). Notably, SLD has emerged as the predominant etiology of cirrhosis in both the European Union and United States, where MASLD and ALD represent the most common subtypes (2). Given that cirrhosis ranks as the 11th leading cause of mortality worldwide (2), the development of early detection strategies for hepatic steatosis carries significant public health implications.

However, translating this imperative into effective community and point-of-care (POC) practice faces an obstacle. The critical need in these settings is for a screening tool that can first and foremost detect the presence of hepatic steatosis, irrespective of its underlying cause (e.g., MASLD, ALD, or mixed etiology). However, most established models are not designed for this etiologically-agnostic task. They often rely on venipuncture for etiology-specific biomarkers and are optimized to identify specific subtypes like MASLD or ALD, causing them to miss other subtypes in real-world populations where etiologies frequently overlap. This gap underscores the need for accessible, non-invasive tools dedicated to the initial, accurate detection of hepatic steatosis itself.

Traditional Chinese Medicine (TCM) posits that internal physiological imbalances manifest through external signs, including complexion, tongue characteristics, and pulse patterns. From the perspective of TCM, the pathological basis of SLD is closely related to phlegm, dampness, blood stasis as well as dysfunction of the liver, spleen and kidney (3), all of which produce observable physical signs. Contemporary research has identified distinguishable tongue and pulse manifestations between SLD patients and healthy controls (4, 5), suggesting potential predictive value of TCM indicators for SLD. Building on these findings, our study aimed to develop a novel hepatic steatosis screening model using TCM diagnostic and common clinical data.

## Materials and methods

### Data source

This research was a secondary analysis of data from “A Real-World Study for the Medical Data of Four Diagnostic Synergies Centered on Tongue Image Data for Major Diseases” (Trial registration: Chinese Clinical Trial Registry, ChiCTR1800018090, registered 29 August 2018). The parent study aimed to establish a real-world clinical database and investigate the association between tongue manifestation and major diseases. It was approved by the ethics committee of the Affiliated Hospital of Chengdu University of Traditional Chinese Medicine (2018-KL050), and all participants provided written informed consent, which included permission for future research use of anonymized data.

### Study population

The study population consisted of participants who underwent routine health checkups at the Affiliated Hospital of Chengdu University of Traditional Chinese Medicine between December 2018 and December 2021. These participants were originally enrolled in the parent study, which also included patients seeking care for chronic diseases. For our analysis, we exclusively focused on the health checkup cohort.

The original inclusion criteria for the parent study were: (1) Age ≥ 18 years and ≤ 75 years; (2) Healthy individuals with no acute or chronic diseases for at least 3 months prior to entering the study, or patients diagnosed with conditions such as hypertension, diabetes, lung cancer, or primary colorectal cancer; (3) Not participating in any other clinical studies; (4) Signed informed consent form by the patient or their immediate family member. Original exclusion criteria were: (1) Individuals with impaired consciousness who cannot express subjective discomfort, or patients with psychiatric disorders; (2) Patients with more than one type of severe secondary progressive malignant tumor or other debilitating diseases; (3) Patients with severe primary diseases affecting one or more major systems (e.g., cardiovascular, hepatic, renal, digestive, or hematopoietic systems); (4) Pregnant or breastfeeding women; (5) Individuals with severe depression or anxiety symptoms; (6) Those currently participating in other clinical trials.

**FIGURE 1:**
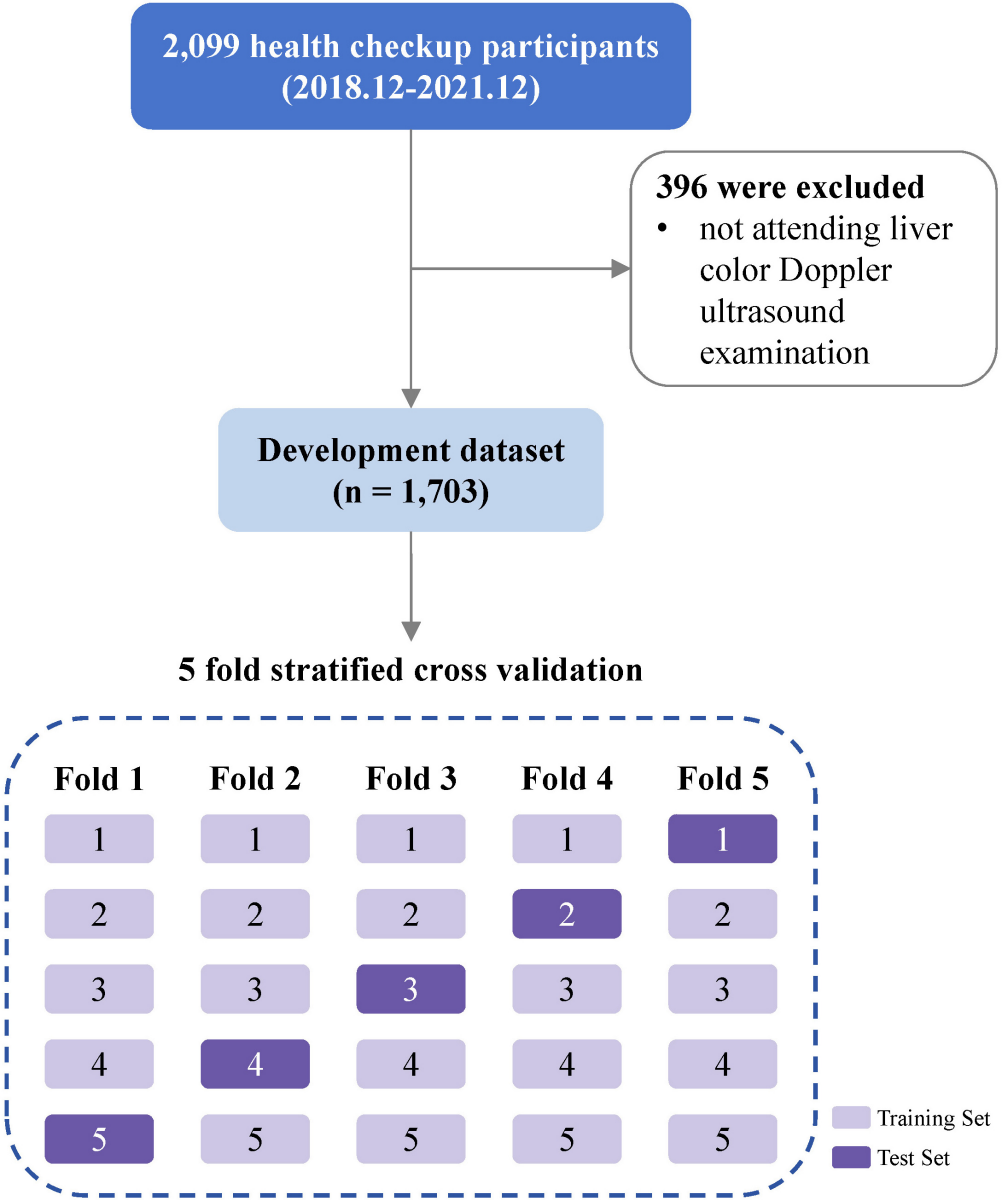
Flow diagram of study design.

Among 2,099 health checkup participants enrolled in the original study, 1,703 participants who have completed the Doppler ultrasound examination of liver and gallbladder were included in this secondary analysis (Figure 1).

### Collection of demographic, anthropometric, and TCM diagnostic data

In the original study, age and gender were filled by the participants on the questionnaire. Height, weight, systolic and diastolic blood pressure were obtained with standardized electronic instruments. Body mass index was calculated based on height and weight. TCM diagnostic data were collected with DAOSH four examinations instrument (Shanghai Food & Drug Administration approval No. 20202200060 for medical devices). It was comprised of face, tongue, pulse manifestation data, TCM constitution data, and TCM-specific symptom data. The detailed measuring process of TCM data is provided in Supplementary Methods.

### Assessment of hepatic steatosis

Hepatic steatosis was diagnosed using Doppler ultrasound. The examinations were assessed by experienced radiologists who were blinded to the study’s aim and the participants’ clinical data. The diagnostic criteria required the presence of at least two out of three characteristic findings on abdominal ultrasonography: diffusely increased echogenicity (“bright”) liver with liver echogenicity greater than kidney or spleen, vascular blurring, and deep attenuation of the ultrasound signal (6).

### Data partitioning and preprocessing

We employed a nested five-fold cross-validation framework to ensure unbiased performance estimation and prevent overfitting. The design consisted of two hierarchical levels. In the outer loop for performance evaluation, the dataset was partitioned into 5 stratified folds preserving outcome variable proportions. Each fold served once as the independent test set while the remaining four formed the training set. Final performance metrics were obtained by aggregating predictions across all five outer test sets. Within each training set, an inner five-fold cross-validation was conducted for model optimization tasks.

Data preprocessing steps included outlier detection, missing value handling, and feature transformation. For optimal predictor selection, we combined recursive feature elimination (RFE) with XGBoost feature importance scoring. The detailed description of data preprocessing is provided in Supplementary Methods. All data preprocessing steps were exclusively fitted on the training set before application to the corresponding test set.

### Model development and evaluation

The aim of our model was to predict the presence of hepatic steatosis. We employed both parametric and non-parametric algorithms to develop the prediction model. Parametric algorithms included logistic regression, linear discriminant analysis, and lasso regression. Non-parametric algorithms included decision tree, random forest, XGBoost, support vector machine, k-nearest neighbor, and Gaussian naive Bayes. Lasso regression performed both predictor selection and modeling, whereas the other algorithms used predictors selected by RFE. Within each outer training fold, the optimal hyperparameters were determined via an inner five-fold cross-validation with grid search. The combination that yielded the highest mean value of the area under the receiver operating characteristic curve (AUC) across these inner validation folds was selected.

The predictive performance of model was evaluated on the outer-loop test sets based on three aspects: Discrimination, calibration, and clinical utility. Discrimination was assessed primarily by the AUC value. Furthermore, the optimal probability threshold was determined by maximizing Youden’s index on the inner-loop validation sets. This threshold was subsequently applied to the outer-loop test sets to calculate threshold-specific metrics, including sensitivity, specificity, positive predictive value (PPV), and negative predictive value (NPV). Calibration was visually examined using calibration curves and assessed by the agreement between predicted probabilities and observed outcomes. Clinical utility was quantified using decision curve analysis (DCA), which estimates the net benefit across a range of clinically relevant probability thresholds. On top of performance, we further employed SHAP (SHapley Additive exPlanations) analysis on the outer-loop test sets to quantify and visualize the contribution of each variable of the selected model.

### Sample size estimation

To ensure adequate statistical power and enhance the generalizability of our findings, we utilized all available data from the database. Sample size estimation was performed based on the method proposed by Riley et al. (7), which accounts for four key parameters: outcome prevalence, number of predictor variables, shrinkage factor, and R^2^. In our study, the prevalence of fatty liver was 25.3%, and we anticipated selecting at most 20 predictor variables. With a target shrinkage factor of 0.9 and an R^2^ of 0.1, the calculated minimum required sample size was 1,698. Our dataset met this requirement, confirming its suitability for robust model development.

### Statistical analysis

Continuous variables were presented as mean ± standard deviation (normal distribution) or median (quartile) (skewed distribution), and categorical variables were presented in frequency or as a percentage. The AUC of two selected models—one parametric and one non-parametric—was compared using the DeLong test, with 95% confidence intervals (CIs) derived from bootstrap resampling (1,000 replicates). Sensitivity and specificity were compared with the McNemar test, whereas bootstrap resampling (also with 1,000 replicates) was applied to compare the positive predictive value, negative predictive value, calibration slope, and calibration intercept across models. A two-sided *p*-value < 0.05 was considered statistically significant. All data were analyzed using Python version 3.7 (Python Software Foundation, Wilmington, DE, United States) with numpy, scipy, pandas, scikit-learn, matplotlib, XGBoost, SHAP libraries, and self-defined functions.

## Results

### General characteristics of participants

Participants diagnosed with hepatic steatosis accounted for 25.3%, and the proportion of males, as well as the values for height, weight, body mass index (BMI), systolic blood pressure (SBP), diastolic blood pressure (DBP), were numerically higher in the hepatic steatosis group than in the non-hepatic steatosis group. Age distributions were similar in the two groups (Table 1).

**TABLE 1:** General characteristics of 1,703 participants stratified by hepatic steatosis status.

Characteristics	Total	Non-hepatic steatosis	Hepatic steatosis
Number (n, %)	1,703	1,272 (74.7%)	431 (25.3%)
Age (years, mean ± SD)	46.9 ± 10.6	46.9 ± 10.8	46.8 ± 9.7
Gender, male (n, %)	1007 (59.1%)	657 (51.7%)	350 (81.2%)
Height (cm, mean ± SD)	162.1 ± 8.6	160.9 ± 8.4	165.6 ± 8.1
Weight (kg, mean ± SD)	64.6 ± 11.9	61.2 ± 10.0	74.7 ± 11.3
BMI (kg/m^2^, mean ± SD)	24.5 ± 3.4	23.6 ± 3.0	27.1 ± 3.0
SBP (mmHg, mean ± SD)	123.0 ± 17.9	121.4 ± 17.9	127.4 ± 16.9
DBP (mmHg, mean ± SD)	76.1 ± 12.1	74.8 ± 12.0	79.9 ± 11.4

SD, standard deviation; BMI, body mass index; SBP, systolic blood pressure; DBP, diastolic blood pressure.

### Selected predictors

After the variable number reached 70, the AUC value increased very slowly as the number went up. So we picked 70 variables from each outer loop training fold and the final predictor set was determined by taking the intersection of variables selected across all outer training folds. Finally, anthropometric indicators weight, BMI, diastolic blood pressure and TCM diagnostic indicators RGB_R of mid tongue, phlegm-dampness constitution score, Lab_A of lip, T4, H5, Lab_A of orbit, T5, HSV_H of nose, a total of 11 variables were selected. AUC-variable number relationships, RFE-selected and LASSO-selected variables (per outer fold), and variance inflation factor analysis are reported in Supplementary Results.

The distribution of selected variables by hepatic steatosis status is shown in Table 2. Large effect sizes (standardized mean differences (SMD) ≥ 0.8) were observed for weight and BMI, and moderate differences (0.5 ≤ SMD < 0.8) were found in HSV_H of nose and Lab_A of orbit, while small effects (0.2 ≤ SMD < 0.5) were noted for DBP, phlegm-dampness constitution score, and RGB_R of mid tongue. In contrast, the differences for T5, Lab_A of lip, H5, and T4 were trivial (SMD < 0.2).

**TABLE 2:** Comparison of selected predictors between hepatic steatosis and non-hepatic steatosis groups.

Predictors	Non-hepatic steatosis	Hepatic steatosis	SMD
Weight (kg, mean ± SD)	61.2 ± 10.0	74.7 ± 11.3	1.305
BMI (kg/m^2^, mean ± SD)	23.6 ± 3.0	27.1 ± 3.0	1.193
HSV_H of nose (none, mean ± SD)	24.2 ± 2.4	22.6 ± 2.1	–0.707
Lab_A of orbit (none, mean ± SD)	7.4 ± 1.2	8.0 ± 1.1	0.501
Diastolic blood pressure (mmHg, mean ± SD)	74.8 ± 12.0	79.9 ± 11.4	0.43
Phlegm-dampness constitution score [none, median (IQR)]	21.9(10.0–37.5)	28.1(15.6–43.8)	0.275
RGB_R of mid tongue (none, mean ± SD)	171.3 ± 7.4	173.0 ± 6.6	0.246
T5 (second, mean ± SD)	0.49 ± 0.16	0.46 ± 0.14	–0.19
Lab_A of lip (none, mean ± SD)	19.7 ± 5.0	18.9 ± 4.6	–0.161
H5 [mm, median (IQR)]	0.43(0.12–0.98)	0.54(0.17–1.08)	0.119
T4 (second, mean ± SD)	0.33 ± 0.06	0.325 ± 0.06	–0.074

SMD, standardized mean differences; SD, standard deviation; BMI, body mass index; IQR, interquartile range.

### Model performance

#### Discrimination of different models

Among three parametric algorithms, AUC values were very close and logistic regression got the highest performance. Among non-parametric algorithms, XGBoost attained the highest AUC value, followed by random forest, support vector machine, Gaussian naive Bayes, k-nearest neighbor, and decision tree (Table 3). Optimal hyperparameters searched for each algorithm are provided in Supplementary Results.

**TABLE 3:** Comparison of the area under the curve (AUC) values for nine algorithms.

Algorithms		AUC	95% CI
Parametric	Logistic regression	0.830	0.809–0.850
	Linear discriminant analysis	0.829	0.808–0.849
	Lasso regression	0.829	0.807–0.850
Non-parametric	XGBoost	0.840	0.818–0.859
	Random forest	0.838	0.816–0.857
	Support vector machine	0.831	0.810–0.851
	Gaussian naive bayes	0.826	0.803–0.847
	K-Nearest neighbor	0.823	0.801–0.845
	Decision tree	0.809	0.786–0.831

AUC, area under curve; CI, confidence interval.

**FIGURE 2:**
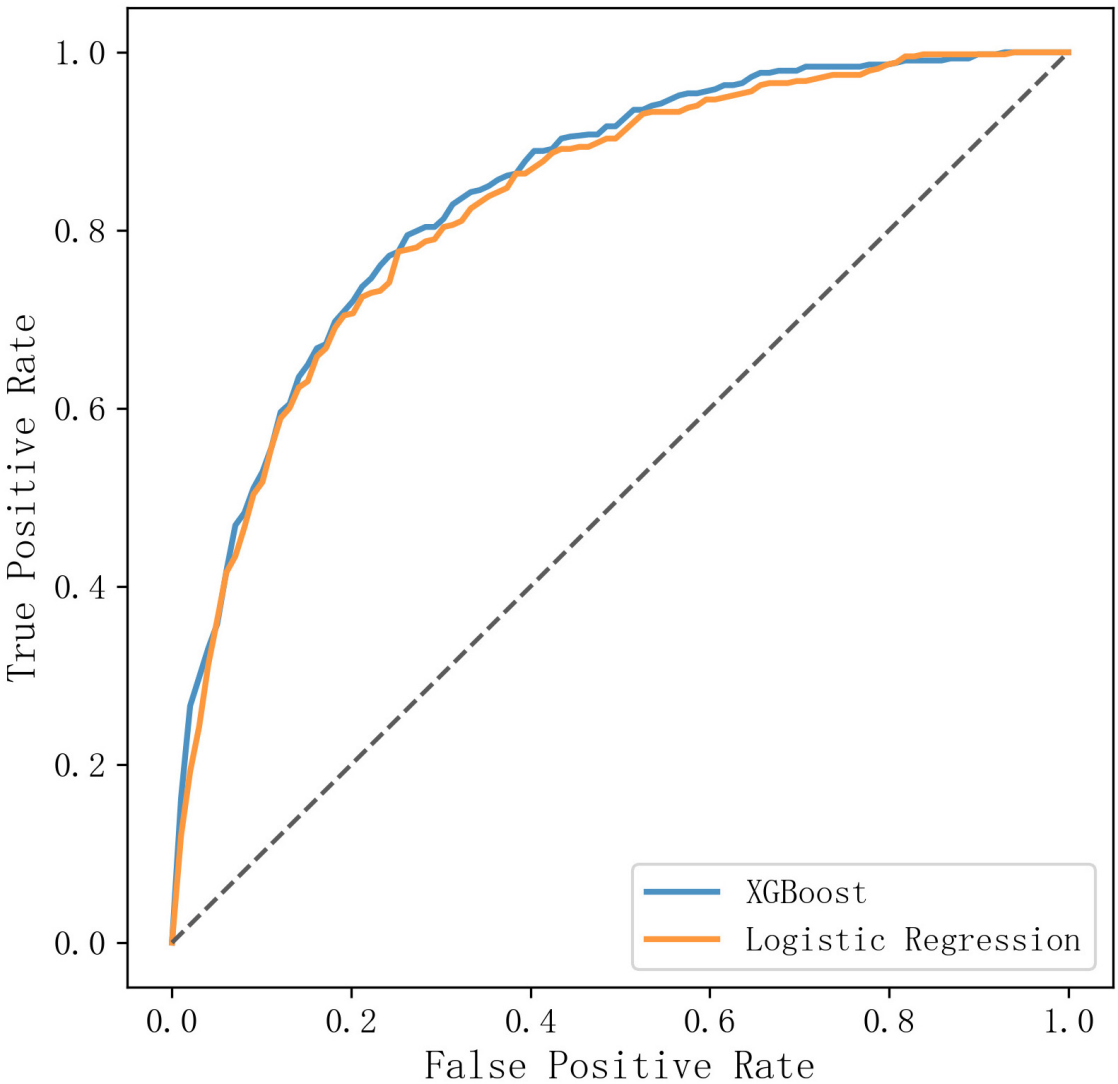
Comparison of ROC curves of XGBoost and logistic regression.

The DeLong test indicated that the AUC of the XGBoost model was statistically significantly higher than that of the logistic regression model (AUC difference = 0.01, *p-*value = 0.012). Graphically, the ROC curves showed that the XGBoost model achieved a marginally higher True Positive Rate (TPR) across various False Positive Rate (FPR) thresholds compared to logistic regression (Figure 2).

The subgroup analysis showed that the predictive performance of the XGBoost model, while generally strong, varied across different participant strata. The model achieved higher AUC in males, younger participants (<60 years), and those without hypertension or diabetes. Although slightly lower AUC values were observed in females, older participants, and individuals with comorbidities, the model maintained acceptable discriminatory power (all AUCs > 0.77) in these clinically relevant subgroups (Table 4). Logistic regression echoed this trend but yielded uniformly lower AUC values in every subgroup (Supplementary Results).

**TABLE 4:** Area under the curve (AUC) for the XGBoost model across subgroups.

Subgroup	Participants (n, %)	AUC	95% CI	*P-*value
**Gender**				
Male	1,007 (59.13%)	0.827	0.801–0.850	<0.001
Female	696 (40.87%)	0.772	0.719–0.819	
**Age**				
<60	1,477 (86.73%)	0.846	0.825–0.867	0.002
≥ 60	226 (13.27%)	0.785	0.713–0.851	
**Hypertension**				
Yes	392 (23.02%)	0.804	0.758–0.849	0.01
No	1,311 (76.98%)	0.846	0.821–0.870	
**Diabetes**				
Yes	215 (12.62%)	0.785	0.718–0.842	0.006
No	1,488 (87.38%)	0.84	0.816–0.863	

AUC, area under curve; CI, confidence interval.

#### Classification performance at the optimal threshold

The classification performance of XGBoost and logistic regression models at their respective optimal thresholds is summarized in Table 5. For XGBoost, the thresholds ranged from 0.204 to 0.288, while for logistic regression, they ranged from 0.226 to 0.301. Although numerical differences favored XGBoost across all metrics, including sensitivity, specificity, and predictive values, none of these differences were statistically significant.

**TABLE 5:** Classification performance of XGBoost and logistic regression models at their optimal probability thresholds.

Metric	XGBoost	Logistic regression	*P-*value
**Optimal threshold**				
- Fold 1	0.249	0.262	
- Fold 2	0.256	0.240	
- Fold 3	0.288	0.249	
- Fold 4	0.263	0.301	
- Fold 5	0.204	0.226	
Sensitivity	0.781	0.762	0.153
Specificity	0.739	0.737	0.856
Positive predictive value (PPV)	0.503	0.495	0.406
Negative predictive value (NPV)	0.909	0.902	0.112

#### Calibration curves of XGBoost and logistic regression

**FIGURE 3:**
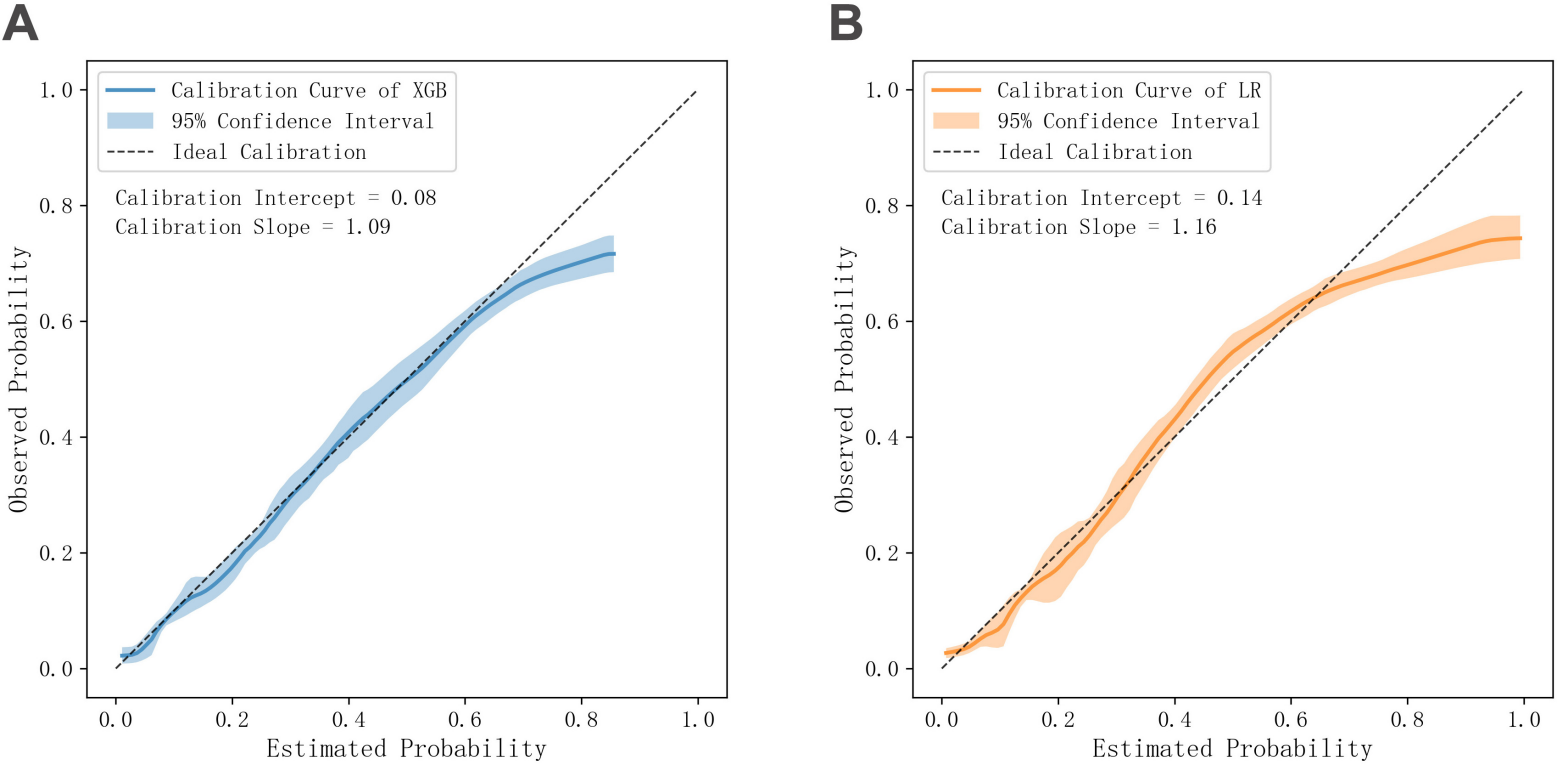
Comparison of calibration curves of XGBoost and logistic regression. **(A)** Calibration curve of XGBoost. **(B)** Calibration curve of logistic regression.

The calibration curve of the XGBoost model demonstrated strong agreement between predicted probabilities and observed outcomes. The calibration intercept was 0.08, indicating minimal overall bias, while the calibration slope of 1.09 suggested near-ideal alignment with slight overestimation for higher probabilities. The 95% confidence interval largely overlapped with the ideal calibration line for predicted probabilities below 0.6. For predicted probabilities above 0.6, the observed probabilities were slightly lower than predicted (Figure 3A). Logistic regression also showed good but slightly inferior calibration, with an intercept of 0.14 and slope of 1.16. While still maintaining reasonable agreement between predicted and observed probabilities, the model exhibited minor systematic deviations—overestimating risk in both low (<0.3) and high (>0.6) probability ranges, with slight underestimation at mid-range probabilities (0.3–0.6) (Figure 3B). The numerical differences in calibration intercepts and slopes between the two models, while favoring XGBoost, were not statistically significant (*p* = 0.499 for intercept; *p* = 0.366 for slope).

#### Decision curves of XGBoost and logistic regression

**FIGURE 4:**
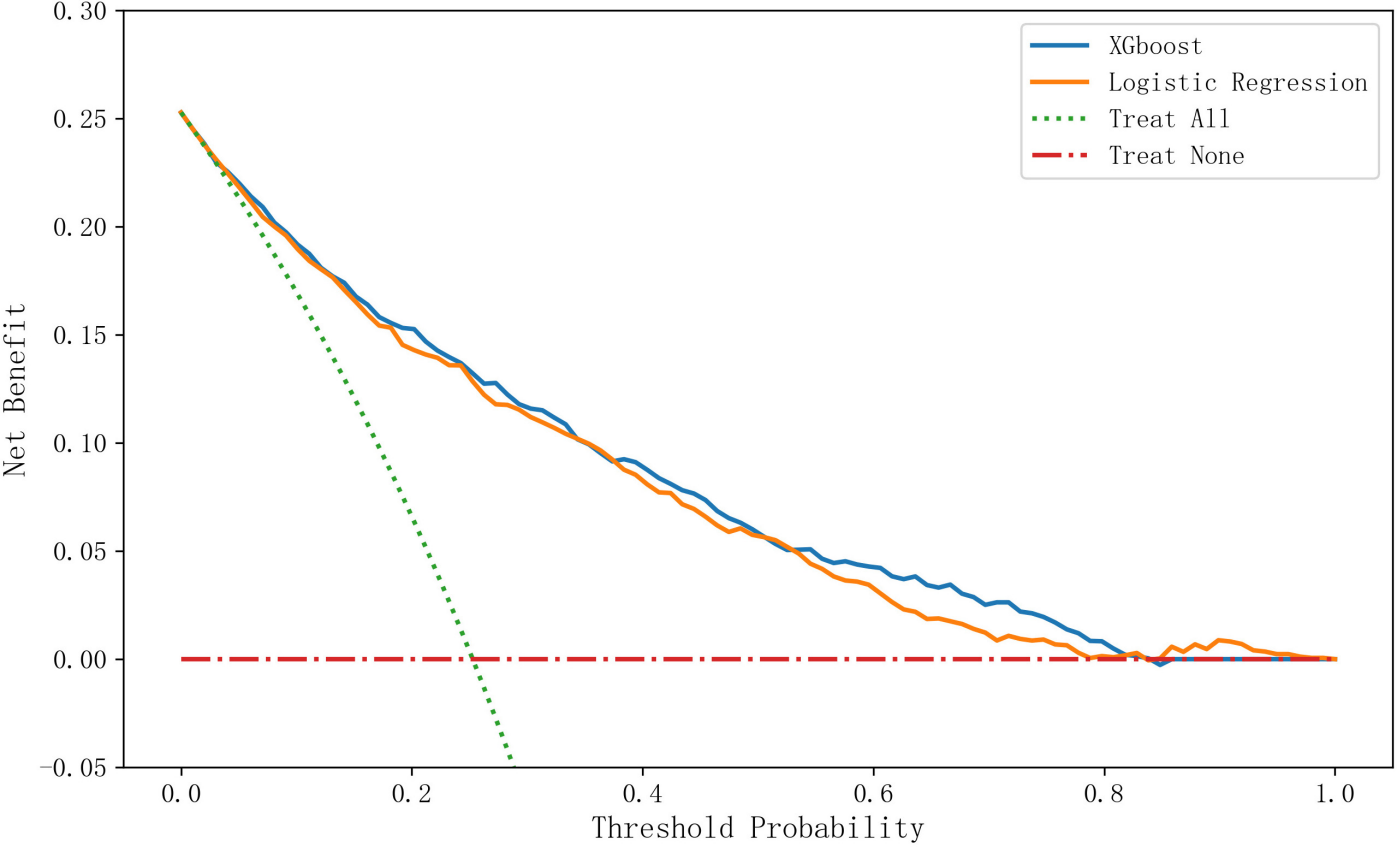
Comparison of decision curves of XGBoost, logistic regression, and different treatment strategies.

As illustrated in Figure 4, the XGBoost model demonstrated superior performance across a broad range of clinically relevant threshold probabilities (0.2–0.8), maintaining the highest net benefit among all decision strategies. The advantage was most pronounced in the 0.6–0.8 threshold range, where XGBoost’s net benefit exceeded that of logistic regression. Notably, a crossover phenomenon occurred beyond the 0.8 threshold probability, with logistic regression yielding marginally higher net benefits.

### Model interpretability

**FIGURE 5:**
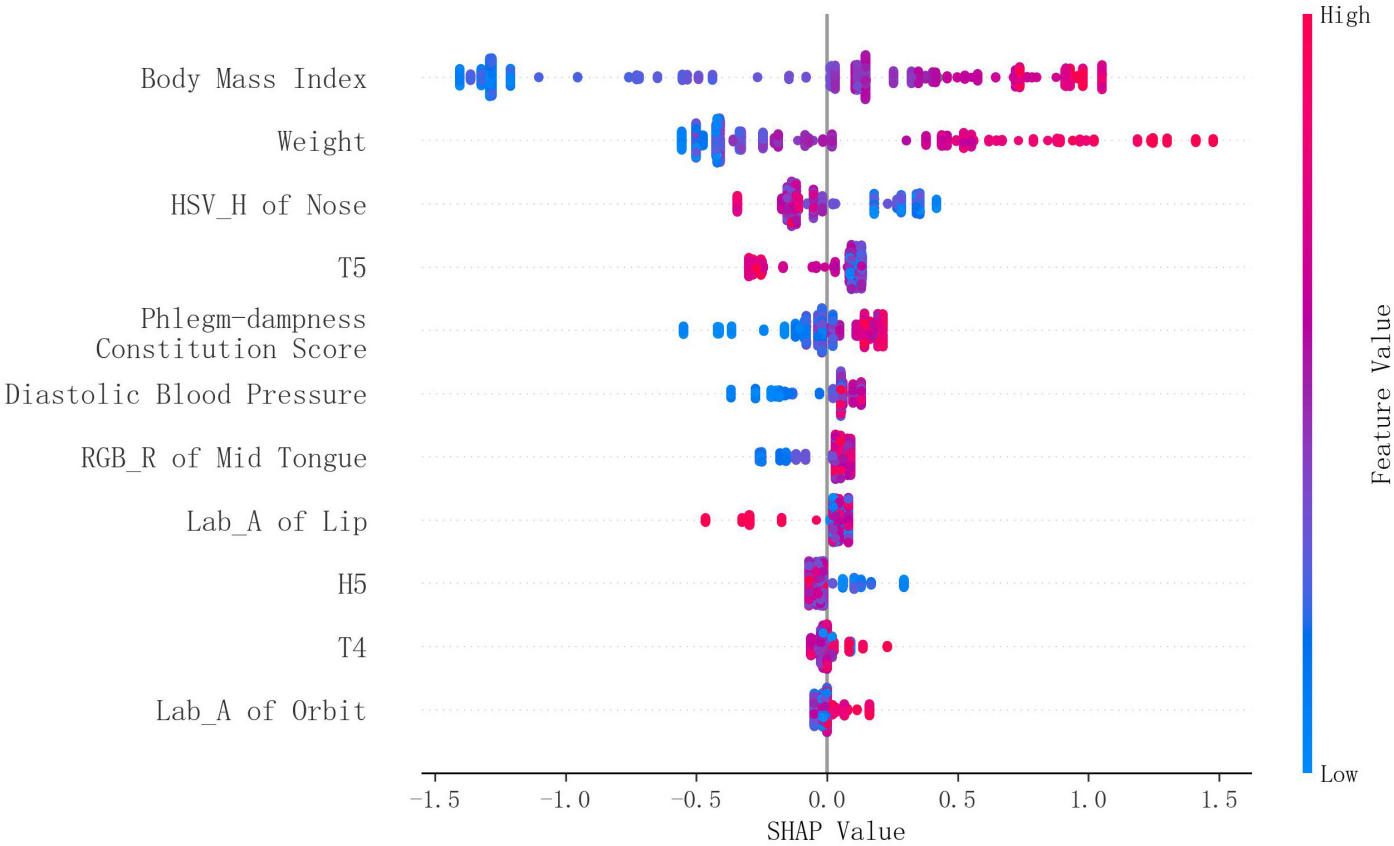
Feature contribution analysis of XGBoost using SHAP plot.

For XGBoost model, the SHAP summary plot revealed that BMI and weight were the most influential predictors, exhibiting the largest absolute SHAP values. Notably, TCM facial feature HSV_H of Nose and pulse feature T5 also demonstrated significant contributions. TCM constitution feature phlegm-dampness constitution score and anthropometric feature diastolic blood pressure exhibited moderate effects on the model’s output. In contrast, localized TCM facial features (e.g., Lab_A of orbit) showed relatively minor contributions (Figure 5).

Logistic regression has inherent interpretability due to its parametric nature. The final logistic regression equation was derived by averaging coefficients across all five folds of cross-validation:


logit(p)=(4.229±0.118)×weight+(3.648±0.163)×

BMI-(1.314±0.247)×HSV_HofNose+(0.923±0.088)×

phlegm⁢constitution⁢score-(0.817±0.081)×T5+

(0.621±0.240)×Lab⁢_⁢A⁢of⁢Orbit+(0.618±0.247)×DBP+

(0.482±0.159)×RGB_RofMidTongue-(0.398±0.091)×

Lab_AofLip-(0.255±0.148)×H5+(0.149±0.136)×

T4-(4.031±0.213)

Similar to XGBoost model, weight, BMI, HSV_H of Nose had relatively large impact on the probability of hepatic steatosis.

## Discussion

In this study, we evaluated nine machine learning algorithms to develop a screening model for ultrasound-detected hepatic steatosis based on purely non-invasive indicators. Among the candidate non-parametric and parametric algorithms, XGBoost and logistic regression demonstrated the best AUC values. Compared to logistic regression, XGBoost exhibited a consistent pattern of minor advantages across all evaluated metrics. It achieved a statistically higher AUC, albeit with a marginal absolute difference ( < 0.02). This trend of nominal improvement was also observed in sensitivity, specificity, predictive values at the optimal threshold, and calibration parameters, although none of these differences reached statistical significance. Decision curve analysis complemented these findings, showing a consistently higher, yet modest, net benefit for XGBoost across most threshold probabilities. Therefore, the choice between the two models depends on the specific clinical context, with XGBoost offering a slight edge in predictive performance and logistic regression providing simplicity and more straightforward interpretability.

Despite the overall robust performance, both XGBoost and logistic regression models exhibited an attenuation in AUC within specific subgroups such as females, older adults ( ≥ 60 years), and individuals with hypertension or diabetes. This phenomenon may be attributed to two non-mutually exclusive factors. First, the reduced sample sizes in these strata likely limited the statistical power to robustly capture the complex mapping between predictors and the outcome. Second, pathophysiological specificity in these populations may render the selected indicators less representative; for instance, hormonal influences on fat distribution in females can diminish the sensitivity of BMI (8), while in older, hypertensive, and diabetic subgroups, the widespread use of medications and the presence of subclinical comorbidities can mask the true metabolic risk, thereby diluting the predictive signal of clinical indicators.

To uncover the drivers of predictions from both the XGBoost and logistic regression models, we leveraged SHAP analysis and interpreted logistic regression coefficients, respectively. Both the SHAP analysis and the logistic regression coefficients identified BMI and weight as dominant contributors. This strongly aligns with established literature linking obesity to hepatic steatosis via mechanisms like insulin resistance and chronic inflammation (9, 10). Despite the correlation between BMI and weight in the model, the VIF analysis showed acceptable multicollinearity (both VIFs < 5, see Supplementary Results), and both the RFE and LASSO methods selected them as predictors, suggesting they provide complementary information for the prediction. Another anthropometric variable diastolic blood pressure played a role as well, exhibiting moderate effects on the model’s output. This is consistent with the known bidirectional association between hypertension and non-alcoholic fatty liver disease (NAFLD, now termed MASLD) (11).

Beyond established clinical indicators, our model incorporated a novel set of digitalized TCM metrics derived from three domains: 1) facial and tongue colors, quantified using HSV (Hue-Saturation-Value), Lab (lightness-chromaticity), and RGB (Red-Green-Blue) models—specifically, HSV_H of nose, Lab_A of lip, Lab_A of orbit, and RGB_R of mid tongue; 2) pulse waveforms, from which parameters T4, T5, and H5 were extracted (H and T denote the height and time values of the pulsation trajectory, respectively); and 3) a phlegm-dampness constitution score calculated from self-reported symptoms, quantifying the degree of phlegm-dampness constitution in TCM. The SHAP analysis and logistic regression coefficients both confirmed the significant predictive role of HSV_H of nose. This association with hepatic steatosis may be mediated through oxidative stress. SLD patients show elevated serum levels of oxidative stress markers such as malondialdehyde and 8-isoprostane (12). These may activate the NF-κB pathway, accelerating dermal fibroblast senescence and promoting lipofuscin deposition in skin tissue (13), potentially leading to nasal skin darkening.

Both T5 and phlegm-dampness constitution score showed relatively high contribution to the prediction. The lower mean T5 value in the hepatic steatosis group, indicating decreased cardiac function, aligns with the existing finding that NAFLD is significantly associated with left ventricular diastolic dysfunction (14). Regarding the phlegm-dampness constitution, its role as a pathological basis of SLD is well-recognized in TCM theory (3), and its epidemiological link to NAFLD has been previously documented (15). Our study further demonstrates the practical utility of this constitution score for hepatic steatosis screening.

Besides, Lab_A of orbit showed a moderate effect size for distinguishing non-hepatic steatosis and hepatic steatosis groups. However, its impact in SHAP analysis was tiny, indicating diminished predictive utility in the presence of stronger predictors. Separately, Lab_A of lip, RGB_R of mid tongue, H5, and T4 were ranked as the least important predictors by SHAP analysis, consistent with their small or trivial univariable effect sizes. Overall, building upon previous evidence that tongue features can assist in predicting NAFLD (16, 17), our study extends these findings by identifying the predictive value of facial and pulse characteristics, as well as TCM constitution scores, thereby broadening the range of TCM tools available for hepatic steatosis screening.

A screening model for hepatic steatosis is crucial for early SLD detection. However, existing models (AUC 0.8–0.89) (18–23) are limited by subtype specificity (e.g., NAFLD, MASLD, ASLD) and reliance on blood tests, restricting their broad use. In contrast, our model avoids subtyping and relies solely on non-invasive predictors to directly identify ultrasound-detected hepatic steatosis. Trained on health check-up data from a health-conscious community subset, it offers a viable path for community screening. While the standardized TCM facial, tongue, and pulse indicators currently require specialized tools, upcoming advances in smartphone color correction and wearable sensor technology are expected to enable at-home measurement in the near future (24, 25).

Apart from model design and practicality, we also strengthened methodological rigor. Our study employed cross-validation to evaluate model performance—a method strongly advocated by Collins et al. (26) to mitigate the limitations of simple data splitting. Unlike previous studies relying on single train-test splits, which discard valuable data and introduce instability due to small test sets, our approach leverages repeated resampling to utilize all available data. This reduces optimism bias from overfitting, providing a more precise performance estimate.

Nevertheless, several limitations of this study should be acknowledged. First, the generalizability of the model is limited. It was developed from a hospital check-up cohort that likely represents individuals with higher socioeconomic status and greater health awareness (27, 28), potentially introducing selection bias. The model has only undergone internal validation and exhibited a moderate decline in performance within key subgroups. Furthermore, its applicability across diverse ethnic and racial groups may be constrained by variations in the manifestation of TCM features. Second, limitations related to the diagnostic standard should be considered. Ultrasound has inherent sensitivity constraints in detecting mild hepatic steatosis, which likely resulted in under-ascertainment of cases and consequently restricts the model’s effectiveness in identifying early-stage disease. Third, the absence of data on established predictors—particularly waist circumference—precluded a head-to-head comparison between our model and conventional, widely used indices such as the Fatty Liver Index (FLI). This gap impedes a direct evaluation of the incremental utility offered by our TCM-based model over existing tools.

Future research should pursue the following directions. First, external validation in community-based settings is necessary to verify the model’s utility in broader public health contexts. Second, future studies should intentionally recruit underrepresented populations such as females, older adults, and individuals with diabetes or hypertension to ensure equitable performance. Third, collecting waist circumference data to enable head-to-head comparison with established tools like the Fatty Liver Index (FLI) and to evaluate the incremental value offered by the TCM-based predictors. Finally, incorporating blood-based biomarkers such as ALT and TG could significantly enhance predictive capacity in high-risk populations. For individuals with conditions like type 2 diabetes or metabolic syndrome, where the pathophysiology of hepatic steatosis is more complex, the addition of biochemical parameters might provide a boost in detection sensitivity.

## Conclusion

TCM facial, tongue, pulse manifestations and phlegm-dampness constitution score can be combined with anthropometric variables to develop a non-invasive screening model for hepatic steatosis identified by ultrasound. Both XGBoost and logistic regression demonstrated strong and comparable performance. The choice between them should be guided by the clinical context—whether inherent interpretability or slight predictive advantage is prioritized.

## Data Availability

The original contributions presented in this study are included in this article/Supplementary material, further inquiries can be directed to the corresponding authors.
