# Supplementary material for: Development of a machine learning model for hepatic steatosis screening using non-invasive Traditional Chinese Medicine diagnostics and clinical variables: a health checkup study with community screening potential
Source: Front Med (Lausanne). 2026 Jan 14;12:1704441. doi: 10.3389/fmed.2025.1704441 (PMC12847276; doi:10.3389/fmed.2025.1704441)
Supplement: Supplementary file 1 [file Presentation_1.pdf]

Supplementary Methods

Measuring Process of TCM Data

Assessment of Facial, Tongue Manifestation

In the early morning under fasting conditions, the participant rinsed the oral cavity and remained calm, refraining from emotional agitation or physical exertion. The examination was conducted under controlled, soft natural illumination. The participant was seated with the chin placed on the dedicated stabilization tray of the DAOSH four examinations instrument. Under the system's standardized lighting - which complies with ISO standards and uses calibrated LED sources along with a high-resolution SLR camera - the participant first closed their eyes for facial image capture. They then gently extended the tongue in a relaxed manner, keeping the dorsal surface flat and the apex natural to ensure full exposure. The operator captured the image only after confirming stable positioning, with real-time verification of image quality to exclude blurring, motion artifacts, or other technical issues. The acquired images were processed by the DS01-A Four-Diagnostic Information Acquisition and Recognition System for automated analysis.

This analysis followed a standardized pipeline to ensure objectivity and reproducibility. System calibration was performed using a 24-color standard checker to maintain color fidelity, and the tongue region was automatically segmented before quantitative parameters (e.g., Lab values) were extracted using established image processing libraries such as OpenCV.

Assessment of Pulse Manifestation

The participant sit upright with the left arm positioned at heart level, maintaining a straight wrist with the palm facing upward while keeping the shoulder and elbow joints relaxed, ensuring an angle of 120° to 135° between the upper arm and forearm. A pulse pillow was placed beneath the dorsal aspect of the wrist for proper support. The operator first located the optimal palpation site (Guan position) on the left radial artery, then secured the single-probe sensor at the point of maximum pulse intensity, applying appropriate pressure while maintaining perpendicular alignment with the skin surface. Throughout the measurement, the participant must remain still to prevent movement artifacts. After powering on the pulse diagnosis instrument, the probe gradually increased pressure until a stable, physiologically representative pulse waveform was obtained, at which point the system automatically recorded the tracing and analyzed pulse characteristics including position, rate, morphology, and amplitude.

Assessment of TCM Symptoms and Constitutions

The electronic version of Wang Qi's Traditional Chinese Medicine Constitution Questionnaire was used to collect participants' symptoms, which contained 60 items corresponding to 60 symptoms, all measured as ordinal variables. Each question offered five response options indicating symptom severity: "None (absolutely not)," "Rarely (a little)," "Sometimes (some)," "Often (quite)," and "Always (very)." Participants self-reported their responses on the Four Diagnostic Instruments according to their situation in the past year, with researchers available to clarify any

questions they did not understand. After completion, the researchers reviewed and verified the participant's symptom reports against the questionnaire results to ensure accuracy. TCM constitution scores were calculated based on the degree of each TCM symptom. The questionnaire is as follows.

Supplementary Table 1 Questionnaire of TCM symptoms

Experience/condition in the past year	No	Slightly	Sometimes	Often	All the time
(1) Were you energetic?	5	4	3	2	1
(2) Did you get tired easily?	5	4	3	2	1
(3) Did you suffer from shortness of breath?	5	4	3	2	1
(4) Did you get palpitations?	5	4	3	2	1
(5) Did you get dizziness easily or become giddy when standing up?	5	4	3	2	1
(6) Did you prefer quietness and do not like to talk?	5	4	3	2	1
(7) Did you feel feeble when talking?	5	4	3	2	1
(8) Did you feel gloomy and depressed?	5	4	3	2	1
(9) Did you get anxious and worried easily?	5	4	3	2	1
(10) Did you feel sensitive, vulnerable or emotionally upset?	5	4	3	2	1
(11) Were you easily scared	5	4	3	2	1

or frightened?					
(12) Did you experience distention in the underarm or breast?	5	4	3	2	1
(13) Did you feel chest or stomach stuffiness?	5	4	3	2	1
(14) Did you sigh for no reason?	5	4	3	2	1
(15) Did your body feel heavy or lethargic?	5	4	3	2	1
(16) Did the palms of your hands or soles of your feet feel hot?	5	4	3	2	1
(17) Did your hands or feet feel cold or clammy?	5	4	3	2	1
(18) Did you feel cold easily in your abdomen, back, lower back or knees?	5	4	3	2	1
(19) Were you sensitive to cold and tend to wear more clothes than others?	5	4	3	2	1
(20) Did your body and face feel hot?	5	4	3	2	1
(21) Did you feel more vulnerable to the cold than others (winter coldness, air conditioners, fans, etc.)?	5	4	3	2	1
(22) Did you catch colds more easily than others?	5	4	3	2	1

(23) Did you sneeze even when you did not have a cold?	5	4	3	2	1
(24) Did you have runny or stuffy nose even when you did not have a cold?	5	4	3	2	1
(25) Did you cough due to seasonal change, temperature change, or unpleasant odor?	5	4	3	2	1
(26) Did you sweat easily when you had a slightly increased physical activity?	5	4	3	2	1
(27) Did you forget things easily?	5	4	3	2	1
(28) Did you have an excessively oily forehead and/or T-zone?	5	4	3	2	1
(29) Were your lips redder than others?	5	4	3	2	1
(30) Did you have allergies? (E.g. medicine, food, odors, pollen, pet dander, or during seasonal or weather change etc.?)	5	4	3	2	1
(31) Did your skin get hives/urticaria easily?	5	4	3	2	1
(32) Did your skin have purpura (purple spots, ecchymosis) due to allergies?	5	4	3	2	1

(33) Did black or purple bruises appear on your skin for no reason?	5	4	3	2	1
(34) Did your skin turn red and show traces when you scratched it?	5	4	3	2	1
(35) Did your skin or lips feel dry?	5	4	3	2	1
(36) Did you have visible capillary/thread veins on your cheeks?	5	4	3	2	1
(37) Did you feel pain somewhere in your body?	5	4	3	2	1
(38) Did you get hot flashes?	5	4	3	2	1
(39) Did your nose or your face feel greasy, oily, or shiny?	5	4	3	2	1
(40) Did you have a dark face or get brown spots easily?	5	4	3	2	1
(41) Did you get acne or sores easily?	5	4	3	2	1
(42) Did you have upper eyelid swelling?	5	4	3	2	1
(43) Did you get dark circles under the eyes easily?	5	4	3	2	1
(44) Did your eyes feel dry and use eye drops?	5	4	3	2	1
(45) Did your lips darker,	5	4	3	2	1

more blue or purple then usual?					
(46) Did you often feel parched and need to drink water?	5	4	3	2	1
(47) Did your throat feel strange (i.e., Like something was stuck or there was a lump in your throat)?	5	4	3	2	1
(48) Did you have bitterness or a strange taste in your mouth?	5	4	3	2	1
(49) Did your mouth feel sticky?	5	4	3	2	1
(50) Was your stomach/belly flabby?	5	4	3	2	1
(51) Did you have lots of phlegm, especially in your throat?	5	4	3	2	1
(52) Did you feel uncomfortable when you drank or ate something cold, or did you avoid drinking or eating something cold?	5	4	3	2	1
(53) Could you adapt yourself to external natural or social environment change?	5	4	3	2	1
(54) Did you suffer from insomnia?	5	4	3	2	1

(55) Did you easily get diarrhea when exposed to a cold or ate (or drank) something cold?	5	4	3	2	1
(56) Did you pass sticky stools and/or feel that your bowel movement is incomplete?	5	4	3	2	1
(57) Did you get constipated easily or have dry stools?	5	4	3	2	1
(58) Did your tongue have a thick coating?	5	4	3	2	1
(59) Did your urethral canal feel hot when you urinated, or did your urine have a dark color?	5	4	3	2	1
(60) Was your scrotum always wet (only for male interviewees)? Was your vaginal discharge yellowish (only for female interviewees)?	5	4	3	2	1

Original scores: Sum up each item's score

Converted scores: [(original scores - items)/(items*4)]* 100

Determination:

Constitution	Condition	Result
Balanced constitution	Converted scores>60	Yes

	The rest constitutions' converted scores all<30	
	Converted scores>60	Basically yes
	The rest constitutions' converted scores all<40	
	Not stratified the conditions above	No
Unbalanced constitution	Converted scores>40	Yes
	Converted scores39~30	Tend to
	Converted scores<30	No

Balanced constitution

Original scores = item 2 + item 7 + item 21 + item 27 + item 8 + item 1 + item 53 + item 54

Converted scores = [((item 2 + item 7 + item 21+ item 27 + item 8 + item 1 + item 53 + item 54)-8)/8*4]*100

Qi-deficiency constitution

Original scores = item 2 + item 3 + item 4 + item 5 + item 22 + item 6 + item 7+ item 26

Converted scores = [((item 2 + item 3 + item 4+ item 5 + item 22+ item 6+ item 7+ item 26)-8)/8*4]*100

Yang-deficiency constitution

Original scores = item 17 + item 18 + item 19 + item 21 + item 22 + item 52 + item 55

Converted scores = [((item 17 + item 18 + item 19 + item 21 + item 22 + item 52 +

item 55)-7)/7*4]*100

Yin-deficiency constitution

Original scores = item 16 + item 20 + item 35 + item 29 + item 57 + item 38 + item 44 + item 46 Converted scores = [((item 16 + item 20 + item 35 + item 29 + item 57 + item 38 + item 44 + item 46)-8)/8*4]*100

Phlegm-dampness constitution

Original scores = item 13 + item 15 + item 50 + item 28 + item 42 + item 49 + item 51 + item 58

Converted scores = [((item 13 + item 15 + item 50 + item 28 + item 42 + item 49 + item 51 + item 58)-8)/8*4]*100

Dampness-heat constitution

Original scores = item 39 + item 41 + item 48 + item 56 + item 59 + item 60

Converted scores = [((item 39 + item 41 + item 48 + item 56 + item 59 + item 60)-6)/6*4]*100

Blood-stasis constitution

Original scores = item 33 + item 36 + item 37 + item 40 + item 43 + item 27 + item45

Converted scores =[((item 33 + item 36 + item 37 + item 40 + item 43 + item 27 + item 45)-7)/7*4]*100

Qi-depression constitution

Original scores = item 8 + item 9 + item 10 + item 11 + item 12 + item 14 + item 47

Converted scores = [((item 8 + item 9 + item 10 + item 11 + item 12 + item 14 + item 47)-7)/7*4]*100

Special diathesis constitution

Original scores = item 23 + item 24 + item 25 + item 30 + item 31 + item 32 + item 34

Converted scores =[((item 23 + item 24 + item 25 + item 30 + item 31 + item 32 + item 34)-7)/7*4]*10

Data Preprocessing

We applied 5-fold stratified cross-validation method to develop and evaluate the predictive model. All preprocessing parameters are estimated solely from the training set and then applied to the test set without recalibration, ensuring no data leakage.

Outliers

Outliers were identified with Tukey's fences method. For each numeric variable, data below the first quartile (Q1) subtracts 1.5 × interquartile range (IQR) or above the third quartile (Q3) pluses 1.5 × IQR were regarded as outliers. After manual examination, all the outliers were considered clinically reasonable, so they were all preserved.

Missing Values

A predefined rule was applied: variables with ⩾30% missing data would be excluded from analysis. In this study, all candidate predictive variables' missing value percentage was below 30%, so no variable was discarded. The missing data were imputed with k-nearest neighbors method (k = 5, euclidean distance) for numeric variables and the mode value for categorical variables.

Variable Encoding and Scaling

TCM-specific symptom data were Likert-scale responses, they were treated as ordered numeric data to preserve the ordinal nature (e.g., 1 = 'not at all' to 5 = 'always'). Nominal variables were encoded using dummy coding to avoid multicollinearity in linear models, and the first category of each variable was set as the reference level. All numeric data were normalized to a [0, 1] range using min-max scaling.

Predictor Selection

We employed a nested cross-validation with recursive feature elimination (RFE) for predictor selection, using extreme gradient boosting (XGBoost) as the core estimator. The procedures were as follows: (1) Outer loop: The data were split into 5-fold training and test sets. Training sets were used for predictor selection and test sets for evaluation of final model performance. (2) Inner loop: Within each outer training fold, a further 5-fold cross-validation was applied (resulting in 25 total sub-training/sub-test splits) to determine the optimal number of predictors. The RFE procedure was implemented with XGBoost, recursively eliminating predictors based on their feature

importance scores derived from the XGBoost model. RFE was initialized with a starting number of 10 predictors and a step size of 5. (3) Predictor selection: For each outer training fold, predictors were selected based on the optimal number identified in the inner loop. (4) Final predictor set: The intersection of predictors selected across all outer training folds was retained as the final predictor set. This conservative approach was chosen to prioritize model stability and parsimony, ensuring the final model would be well-powered given the study's sample size.

Supplementary Results

Predictor Selection Process by RFE and LASSO

The trend of AUC values over number of variables is shown in Supplementary Figure 1. After the variable number reached 70, the AUC value increased very slowly as the number went up. So we picked 70 variables from each outer loop training fold and the final predictor set was determined by taking the intersection of variables selected across all outer folds.

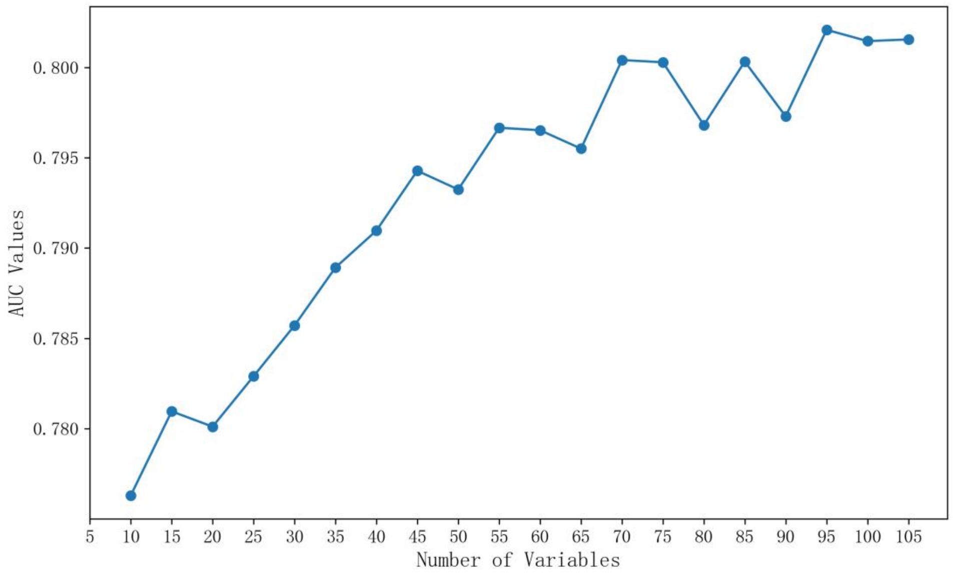

Supplementary Figure 1 AUC-variable number relationships

Finally, anthropometric indicators weight, BMI, diastolic blood pressure and TCM diagnostic indicators RGB_R of mid tongue, phlegm-dampness constitution score, Lab_A of lip, T4, H5, Lab_A of orbit, T5, HSV_H of nose, a total of 11 variables were selected by RFE. The introduction of TCM features is as follows.

Supplementary Table 2 Brief introduction of selected TCM features

TCM Feature	Measurement Method	Anatomical Region	Explanation
RGB_R of mid tongue	Tongue Diagnostic Device	Middle tongue	RGB is a color model that creates a wide range of colors by adding together different intensities of Red, Green, and Blue light. RGB_R of mid tongue means the R value of mid tongue. A high R value in a tongue image primarily indicates the presence of a red color tone, which suggests heat or inflammation in the body.
Lab_A of lip	Facial Diagnostic Device	Lip	The Lab color model is designed to approximate

			human vision, representing colors based on how they are perceived rather than how they are created by devices. Lab_A of lip means the A value of lip. A low A value suggests lips that are paler or less red. This might be interpreted as a sign of “deficiency” (like blood or energy deficiency) or “cold”.
Lab_A of orbit	Facial Diagnostic Device	Orbit	Lab_A of orbit means the A value of orbit. A high A value suggest increased blood flow or capillary presence in the area. This might be interpreted as a sign of heat, inflammation, or stagnation (e.g., related to the liver or sleep patterns)
HSV_H of nose	Facial Diagnostic Device	Nose	HSV is a color model that defines colors by Hue,

			Saturation, and Value for intuitive color selection. HSV_H of nose means the H value of nose. Abnormal H values may indicate localized skin color changes, potentially linked to blood circulation issues, inflammation, or metabolic patterns.
H5	Pulse Diagnostic Device	Radial artery	The dicrotic wave amplitude refers to the vertical height from the peak of the dicrotic wave to a baseline parallel line drawn from the lowest point of the dicrotic notch. This amplitude primarily reflects the elasticity (compliance) of the large arteries and the function of the aortic valve. When arterial compliance

			decreases, h5 is reduced. In cases of aortic valve sclerosis or insufficiency, h5 may become zero (where the peak of the dicrotic wave is level with the lowest point of the dicrotic notch) or even negative (where the peak of the dicrotic wave falls below the level of the dicrotic notch’s lowest point).
T4	Pulse Diagnostic Device	Radial artery	The time interval from the starting point of the pulse wave to the dicrotic notch. T4 corresponds to the systolic period of the left ventricle.
T5	Pulse Diagnostic Device	Radial artery	The time interval from the dicrotic notch to the endpoint of the pulse wave. T5 corresponds to the diastolic

			period of the left ventricle.
Phlegm-dampness constitution score	Questionnaire	/	Phlegm-dampness constitution score quantifies an individual's tendency to accumulate dampness and phlegm in the body. A higher total score indicates a more pronounced phlegm-dampness constitution, which is believed to predispose individuals to conditions like obesity, digestive issues, and fatigue.

Without the use of RFE method, LASSO regression selected predictors based on its own algorithm. Variables selected by LASSO in different folds are listed in Supplementary Table 3.

Supplementary Table 3 Predictors selected by LASSO

Fold Number	Selected Predictors
1	HSV_H of left face, HSV_H of right face, HSV_H of nose, HSV_H of tip tongue fur, Weight, BMI, Sensation of chill, Sweat easily without reason, Constipation, Excessive phlegm,

	Excessive sebum secretion on the forehead, Abdominal obesity, Yellow and white mixed tongue fur, Yellowish vaginal discharge, Pulse shape_abc type, Phlegm-dampness constitution, Blood stasis constitution, Yellowish face, Damp scrotum, Obesity, Male.
2	HSV_S of tongue root, Weight, BMI, Sensation of chill, Sweat easily without reason, Constipation, Excessive sebum secretion on the forehead, Abdominal obesity, Bluish-purple ecchymosis, Prone to mental tension, sneeze even when not having a cold, Irregular pulse rhythm, Teeth marked tongue, Yellow and white mixed tongue fur, Pulse shape_abc type, Qi-insufficiency constitution, Phlegm-dampness constitution, Blood stasis constitution, Yellowish face, Damp scrotum, Male.
3	HSV_H of orbit, HSV_H of nose, HSV_S of tongue root, HSV_H of left tongue fur, HSV_H_11 of tip tongue fur, Weight, BMI, Fatigue, Sensation of chill, Sweat easily without reason, Having diarrhea after eating or drinking cold food, Constipation, Cold sensitivity in the epigastric region, back, or waist/knees, Excessive sebum secretion on the forehead, Abdominal obesity, Bluish-purple ecchymosis, Prone to nervousness and anxiety, Teeth marked tongue, Yellow and white mixed tongue fur, Yellowish vaginal discharge, Pulse shape_abc, Qi deficiency constitution, Phlegm-dampness constitution, Blood stasis constitution, Yellowish

	face, Damp scrotum, Moderate body shape, Male, Pulse strength.
4	HSV_H of orbit, HSV_S of tongue root, Weight, BMI, Sensation of chill, Sweat easily without reason, Cold hands and feet, Constipation, Frequent dry mouth and throat, Excessive sebum secretion on the forehead, Abdominal obesity, Bluish-purple ecchymosis, Prone to nervousness and anxiety, Yellow and white mixed tongue fur, Yellowish vaginal discharge, Pulse shape_abc, Red lips, Phlegm-dampness constitution, Blood stasis constitution, Yellowish face, Damp scrotum, Male.
5	HSV_H of orbit, HSV_H of left tongue fur, Weight, BMI, Sensation of chill, Sweat easily without reason, Constipation, Dry eye sensation, Excessive sebum secretion on the forehead, Abdominal obesity, Burning sensation during urination with dark-colored urine, Bluish-purple ecchymosis, Bodily pain, Sneezing without cold symptoms, Yellow and white mixed tongue fur, Yellowish vaginal discharge, Pulse shape_abc, Qi-deficiency constitution, Phlegm-dampness constitution, Blood stasis constitution, Yellowish face, Yin-deficiency constitution, Damp scrotum, Moderate body shape, Male, Pulse strength.

Optimal Hyperparameters Searched for Each Algorithm

Optimal hyperparameters were discovered though 5 fold cross-validation and grid search. Nine algorithms' hyperparameters are listed in Supplementary Table 4.

Supplementary Table 4 Optimal Hyperparameters of Each Algorithm

Algorithms	Hyperparameters
Logistic regression	Not applicable
Linear discriminant analysis	Not applicable
Lasso regression	C=0.16, penalty='l1', solver='saga',max_iter=1000
XGBoost	max_depth=1,min_child_weight=9,colsample_bytree=0.8,subsample=1, eta=0.01, n_estimators=1092
Random forest	criterion='entropy', max_depth=5, max_features='log2', min_samples_leaf=5, min_samples_split=5, n_estimators=250
Support vector machine	C=0.01, kernel='linear'
Gaussian naive Bayes	var_smoothing=1e-10
K-nearest neighbor	metric='manhattan', n_neighbors=30, p=1, weights='distance'
Decision tree	criterion='gini', max_depth=3, max_leaf_nodes=7, min_samples_leaf=3, min_samples_split=2

Variance Inflation Factor Analysis

The Variance Inflation Factor (VIF) was calculated to assess multicollinearity. As shown in Supplementary Table 5, all VIF values were below 5, indicating that multicollinearity is acceptable in our model.

Supplementary Table 5 Variance Inflation Factor of each selected variable

Fold	Variable	Variance Inflation Factor (VIF)
	Weight	3.84
	RGB_R of mid tongue	1.07
	Phlegm constitution score	1.04
	Lab_A of lip	1.36
	T4	1.11
1	BMI	3.29
	Diastolic blood pressure	1.22
	H5	1.14
	Lab_A of orbit	2.10
	T5	1.03
	HSV_H of nose	2.27
	Weight	3.78
	RGB_R of mid tongue	1.06
	Phlegm constitution score	1.04
	Lab_A of lip	1.41
	T4	1.14
2	BMI	3.28
	Diastolic blood pressure	1.21
	H5	1.15
	Lab_A of orbit	2.11
	T5	1.05
	HSV_H of nose	2.23
	Weight	3.79
3	RGB_R of mid tongue	1.06
	Phlegm constitution score	1.03

	Lab_A of lip	1.44
	T4	1.12
	BMI	3.34
	Diastolic blood pressure	1.23
	H5	1.16
	Lab_A of orbit	2.06
	T5	1.05
	HSV_H of nose	2.16
	Weight	3.70
	RGB_R of mid tongue	1.05
	Phlegm constitution score	1.05
	Lab_A of lip	1.47
	T4	1.12
4	BMI	3.32
	Diastolic blood pressure	1.22
	H5	1.14
	Lab_A of orbit	2.13
	T5	1.05
	HSV_H of nose	2.22
	Weight	3.59
	RGB_R of mid tongue	1.06
	Phlegm constitution score	1.04
5	Lab_A of lip	1.45
	T4	1.15
	BMI	3.19
	Diastolic blood pressure	1.23

H5	1.15
Lab_A of orbit	2.25
T5	1.05
HSV_H of nose	2.37

Subgroup analysis of XGBoost and Logistic Regression

The subgroup analysis of the logistic regression model revealed that the predictive performance varied across different participant strata. The model achieved higher AUC in males, younger participants (< 60 years), and those without hypertension or diabetes. Although slightly lower AUC values were observed in females, older patients, and individuals with comorbidities, the model maintained acceptable discriminatory power in these clinically relevant subgroups (Supplementary Table 6).

Supplementary Table 6 AUC of the Logistic Regression model in different subgroups

Subgroup	Participants (n, %)	AUC	95% CI	*P* value
Gender				
Male	1007 (59.13%)	0.819	0.791 - 0.843	<0.001
Female	696 (40.87%)	0.759	0.707 - 0.808	
Age				
＜60	1477 (86.73%)	0.839	0.817 - 0.861	<0.001
⩾60	226 (13.27%)	0.76	0.684 - 0.832	
Hypertension				
Yes	392 (23.02%)	0.785	0.737 - 0.832	<0.001

No	1311 (76.98%)	0.839	0.814 - 0.863	
Diabetes				
Yes	215 (12.62%)	0.76	0.692 - 0.823	<0.001
No	1488 (87.38%)	0.831	0.806 - 0.856	

Sensitivity and specificity of the XGBoost model across different subgroups are summarized in Supplementary Table 7. An analysis of model fairness, using sensitivity and specificity as key metrics, revealed performance disparities. In the gender subgroup, the model demonstrated high sensitivity (0.872) and moderate specificity (0.608) in males, whereas in females, sensitivity was substantially lower (0.383) despite higher specificity (0.881), indicating a potential bias against female patients. Within the age subgroups, sensitivity and specificity were comparable, suggesting no significant age-related bias. Notably, the model exhibited lower specificity in patients with hypertension (0.645 vs. 0.764) and particularly in those with diabetes (0.558 vs. 0.757), highlighting a tendency for more false positives in these comorbid subgroups. The *p*-values for most comparisons were statistically significant, further supporting the existence of these performance variations across populations. Sensitivity and specificity of Logistic regression model in different subgroups showed similar trends (Supplementary Table 8).

Supplementary Table 7 Sensitivity, Specificity of the XGBoost model in different subgroups

Subgroup	Prevalence (n, %)	Sensitivity	*P* value	Specificity	*P* value
Gender					

Male	350 (34.76%)	0.872	<0.001	0.608	<0.001
Female	81 (11.64%%)	0.383		0.881	
Age					
＜60	385 (26.07%%)	0.795	0.066	0.740	1.000
⩾60	46 (20.35%%)	0.667		0.739	
Hypertension					
Yes	392 (34.69%)	0.804	0.489	0.645	<0.001
No	1311 (22.50%)	0.769		0.764	
Diabetes					
Yes	215 (47.91%)	0.827	0.241	0.558	<0.001
No	1488 (22.04%)	0.766		0.757	

Supplementary Table 8 Sensitivity, Specificity of the Logistic Regression model in different subgroups

Subgroup	Prevalence (n, %)	Sensitivity	*P* value	Specificity	*P* value
Gender					
Male	350 (34.76%)	0.861	<0.001	0.585	<0.001
Female	81 (11.64%%)	0.333		0.900	
Age					
＜60	385 (26.07%%)	0.782	0.011	0.734	0.609
⩾60	46 (20.35%%)	0.604		0.755	
Hypertension					
Yes	392 (34.69%)	0.753	0.573	0.768	<0.001

No	1311 (22.50%)	0.783		0.618	
Diabetes					
Yes	215 (47.91%)	0.739	0.056	0.757	<0.001
No	1488 (22.04%)	0.837		0.531	
